# Tendency to Physical Degeneration

**Published:** 1891-07

**Authors:** 


### TENDENCY TO PHYSICAL DEGENERATION.

Decided tendencies to physical degeneration in the American people are commented upon by a recent medical writer. This degeneracy he attributes to unnatural conditions, concomitants of our civilization. Recent studies of our population indicate a rapid growth of the nervous and intellectual organism at the expense of the muscular and vital system. The extraordinary mental activity of the American people has long been a matter of national pride. Experience and observation show, however, that where this is purchased at the price of physical degeneration, it should be a matter more of anxious concern than of patriotic complacency. A quickened cerebral and nervous system makes heavier draughts upon bodily strength, and if these are not met by a corresponding increase of nutrition they will undermine the action of the whole system.

Among the causes for a decline in physical vigor, are noted the increased population of our cities and towns, and the relative decrease in farming population. The close confinement of city occupations and the lack of proper sanitary regulations, have made city life a serious absorbent of muscular and vital energy. Nervous and mental activity are greatly quickened in these centers, but the accompanying physical decline has been a matter of common as well as scientific observation. Added to this cause, are the artificial influences of education, which tell even more fatally upon the female than the male constitution. Each generation of mothers imparts a decreasing measure of vitality to their offspring.

